# CstF-64 and 3′-UTR *cis*-element determine Star-PAP specificity for target mRNA selection by excluding PAPα

**DOI:** 10.1093/nar/gkv1074

**Published:** 2015-10-22

**Authors:** Divya T. Kandala, Nimmy Mohan, Vivekanand A, Sudheesh AP, Reshmi G, Rakesh S. Laishram

**Affiliations:** Cancer Research Program, Rajiv Gandhi Centre for Biotechnology, Trivandrum 695014, India

## Abstract

Almost all eukaryotic mRNAs have a poly (A) tail at the 3′-end. Canonical PAPs (PAPα/γ) polyadenylate nuclear pre-mRNAs. The recent identification of the non-canonical Star-PAP revealed specificity of nuclear PAPs for pre-mRNAs, yet the mechanism how Star-PAP selects mRNA targets is still elusive. Moreover, how Star-PAP target mRNAs having canonical AAUAAA signal are not regulated by PAPα is unclear. We investigate specificity mechanisms of Star-PAP that selects pre-mRNA targets for polyadenylation. Star-PAP assembles distinct 3′-end processing complex and controls pre-mRNAs independent of PAPα. We identified a Star-PAP recognition nucleotide motif and showed that suboptimal DSE on Star-PAP target pre-mRNA 3′-UTRs inhibit CstF-64 binding, thus preventing PAPα recruitment onto it. Altering 3′-UTR *cis*-elements on a Star-PAP target pre-mRNA can switch the regulatory PAP from Star-PAP to PAPα. Our results suggest a mechanism of poly (A) site selection that has potential implication on the regulation of alternative polyadenylation.

## INTRODUCTION

All eukaryotic mRNAs except for those encoding histones have a poly (A) tail at the 3′-end which confers stability and is required for export and translation of the mRNA (1–4). Polyadenylation is carried out by a 3′-end processing complex in a two-step reaction - cleavage at the 3′-UTR followed by the addition of poly (A) tail (∼250 adenosine nucleotides) to the cleaved RNA in the nucleus (1,4–7). Mass spectrometry analysis identified ∼85 protein factors associated in the 3′-end processing complex (8,9). Some of the key factors in the complex include cleavage and polyadenylation specificity factor, CPSF (comprised of 160, 73, 100, 30 kilodaltons, hFIP1 and WDR33 subunits) involved in AAUAAA signal recognition and cleavage (10–18), cleavage stimulatory factor, CstF that binds the U/GU rich downstream sequence element (DSE) and helps assemble a stable cleavage complex that recruits poly (A) polymerase (PAP)(19–23), Cleavage factor Im and IIm that interacts with PAP (24–28), scaffolding protein symplekin (29,30) and poly (A) binding protein (PABPN1) that stabilises the poly (A) tail (31,32). PAP is also required for the cleavage reaction in a yet unidentified mechanism (4,33). Canonical PAPs (PAPα/γ) are responsible for the general polyadenylation of nuclear pre-mRNAs (34–39). Recent studies on another nuclear PAP, Star-PAP have shown that PAPs specifically target select mRNAs for polyadenylation (40,41), however the mechanism of target mRNA selection is still unknown.

Star-PAP (*S*peckle *t*argeted PIPKI*α r*egulated *p*oly (*A*) *p*olymerase) is a nuclear non-canonical PAP regulated by lipid second messenger phosphatidyl-inositol-4,5-bisphosphate (PI4,5P_2_)(41). As the name suggests Star-PAP is associated with and regulated by the enzyme phosphatidyl-inositol-4-phosphate-5-kinase type Iα (PIPKIα) that synthesises nuclear PI4,5P_2_. Star-PAP polyadenylates select subset of mRNAs in the cell involved in oxidative stress response, apoptosis and cancer (40–42). Star-PAP activity is stimulated by oxidative stress treatment of the cell and increases over 10-fold upon PI4,5P_2_ binding (40,41). Star-PAP associates with co-activators PIPKIα, and kinases casein kinase I (CKI) α/ϵ and protein kinase Cδ (PKCδ) that in turn regulates Star-PAP function (41–44). Star-PAP is classified as a non-canonical PAP due to its sequence similarity, however, it shows functional similarity to a canonical PAP (2). Yet, Star-PAP follows a distinct mechanism for cleavage and polyadenylation. Star-PAP directly binds the target UTR RNA upstream of poly (A) signal (PAS) and recruits the cleavage factor CPSF-160 and CPSF-73. This is in contrast to PAPα which is recruited by the interaction with CPSF and CstF that assembles a stable cleavage complex at the poly (A) site (4,33,40).

One crucial question that remains unanswered is the specificity of PAPs for target mRNA selection. Star-PAP selects pre-mRNA UTRs for polyadenylation, and, interestingly, Star-PAP target mRNAs are independent of PAPα (40,41). Even under Star-PAP knockdown condition in the cell, PAPα does not process Star-PAP regulated transcripts and vice versa. However, the mechanism of this PAP specificity is still unclear. Star-PAP directly binds pre-mRNA, yet the clear binding motif is not known (40). *In vitro* footprints of Star-PAP on *HMOX1* and *BIK* UTRs have shown a GC-rich sequence (42). A recent *in vitro* ‘RNA compete’ analysis using GST-Star-PAP to pulldown specific nucleotides from a pool of random oligos indicated an enrichment of -AUA- containing sequence (45). Interestingly, this motif is also present in the Star-PAP footprints so far identified, yet the significance of this motif in Star-PAP *in vivo* mRNA binding is not understood (40,42). Moreover, Star-PAP target UTRs such as *HMOX1, BIK* or *NQO1* UTR have canonical AAUAAA signal and cleavage site. Therefore, it is unclear how PAPα is unable to process or is excluded from the Star-PAP target pre-mRNAs. Sequence analysis of Star-PAP target genes has indicated a low U content in the DSE (42) suggesting a possible role of CstF-64 in determining PAP specificity.

In this paper, we investigate the mechanism of Star-PAP specificity and how PAPα is excluded from Star-PAP target mRNAs. We showed that Star-PAP competes with PAPα for binding to CPSF-160 but with a preference to Star-PAP. We identified a Star-PAP binding nucleotide motif with a core -AUA- element upstream of PAS that confers Star-PAP specificity. Finally, we demonstrated that suboptimal DSE at the Star-PAP target UTRs prevents CstF-64 binding and renders PAPα unable to be recruited even in absence of Star-PAP. Introduction of a U-rich sequence at the DSE followed by mutation of Star-PAP recognition (-AUA-) motif on a Star-PAP target mRNA switches the regulatory PAP from Star-PAP to PAPα. Our results demonstrate a mechanism of Star-PAP specificity for target UTR through specific poly (A) site selection that has possible implications on the regulation of alternative polyadenylation (APA).

## MATERIALS AND METHODS

### Cell culture and transfections

Human embryonic kidney 293 and HeLa cell lines were obtained from American Type Culture Collection and maintained in Dulbecco's modified Eagle's medium with 10% FBS and penicillin/streptomycin (50 U/ml) at 37°C in 5% CO_2_. siRNA oligos were transfected into HEK 293 cells using Oligofectamine (Invitrogen) reagent and plasmid DNAs using Lipofectamine (Invitrogen) as per manufacturer's instructions. Cells were harvested 48 h post transfection. RNAi oligos used for knockdown are shown in supplementary information.

### Protein purifications

Recombinant His-Star-PAP, -PAPα and -CstF-64 were purified from pET28b constructs. The recombinant protein constructs were overexpressed in *BL21(DE3)* by inducing with 1 mM isopropyl thio-β-D-galactoside (IPTG) at 18°C and purified using Ni-NTA affinity chromatography as described previously (40,46). All the procedures were carried out at 4°C. Proteins were concentrated with poly ethylene glycol (PEG 20000 mw), snap frozen and stored in –80°C.

### GST-pulldown assay and immunoblot analysis

GST-Star-PAP or -PAPα was immobilised on pre-equilibrated glutathione-sepharose beads (Invitrogen) overnight at 4°C using over-expressed *Escherichia coli (BL21)* lysates and GST-pulldown experiments were carried out from HEK 293 cell lysates as described earlier (40). Increasing amounts of recombinant His-Star-PAP (0–200 nM) or -PAPα (0–200 nM) were added to the pulldown reaction. Buffers were supplemented with protease inhibitor cocktail (Roche), DNase I and RNase A to rule out interactions through nucleic acids. The inputs show 10% of the lysates used for pulldown. Immunoblottings were carried out as described earlier (40). Antibodies used are given in supplementary information.

### 3′-RACE assay and 3′-end cleavage measurement

Total RNAs were isolated from HEK 293 cells using RNAeasy mini Kit (Qiagen). 3′-RACE assays were carried out using the 3′-RACE system (Invitrogen) according to manufacturer's instructions with 2 μg of total RNA. The RACE products were confirmed by sequencing. For measurement of cleavage efficacy, uncleaved mRNA levels were measured by quantitative real time PCR (qRT-PCR) using a pair of primers across the cleavage site as described earlier (41). The non-cleaved messages were expressed as fold-change over the total mRNA. The gene specific primers used in 3′-RACE and cleavage assays are shown in supplementary information.

### RNA immunoprecipitation (RIP)

RNA immunoprecipitation experiments were carried out after cross linking proteins and RNA with 1% formaldehyde in HEK 293 cells using specific antibodies against CPSF-160, CstF-64, RNA Pol II, Star-PAP and PAPα as described previously (40). The gene specific primers used for detecting *BIK, NQO1, GAPDH* UTRs and antibodies used are listed in the supplementary information.

### Quantitative real-time PCR (qRT-PCR)

qRT-PCR was carried out in a CFX98 multi-colour system (Bio-Rad) with SYBR Green Supermix as described previously (42) from total RNA reverse transcribed using RT-PCR kit (Biorad). Single-product amplification was confirmed by melting-curve analysis, and primer efficiency was near 100% in all experiments. Quantification is expressed in arbitrary units, and target mRNA abundance was normalised to the expression of GAPDH with the Pfaffl method. All qRT-PCR results were representative of at least three independent experiments (*n* > 3). Primers used for qRT-PCRs are indicated in supplementary information.

### RNA EMSA experiment

Uniformly radiolabelled *BIK, NQO1* or control *GCLC* UTR RNAs were prepared by *in vitro* transcription using corresponding DNA construct pTZ-*NQO1* or pTZ-*BIK* encompassing corresponding UTR regions (downstream sequence from PAS for CstF-64 binding assay; and upstream sequence from PAS for Star-PAP binding assays) under T7 promoter. EMSA experiments were carried out as described earlier (40) in a 20 μl EMSA-binding buffer (10 mM Tris-HCl, pH-7.5, 1 mM EDTA, 50 mM NaCl, 0.5 mM MgCl_2_, 1 mM DTT) accompanied with 1 μg/ml bovine serum albumin, 50% glycerol in the presence of 0.5 nM radiolabelled RNA and increasing His-Star-PAP (5 to 50 nM) or -CstF-64 (20–200 nM) at RT. For competition experiments 100-fold excess of each non-radiolabelled RNAs were added in the EMSA reaction.

### *In silico* sequence analysis

Microarray data for Star-PAP knockdown from HEK 293 cells (41) were analysed by in-house perl scripts for the occurrence of -AUA- motif from the down regulated genes in the putative Star-PAP binding region (40,42). A cut-off of fold-change >1.8 was used to select genes showing significant down regulation (considered as Star-PAP targets), and sequences similar to AAUAAA signal were excluded from the analysis. The significance combinatorics of each motif (5-mer or 7-mer) were assessed by Fisher's exact test using FIMO (http://meme-suite.org/tools/fimo)(47) with a cut-off *P*-value of <0.05 (*t*-test). Significant 5-mers were mapped over 7-mer sequences in order to increase motif confidence. The corresponding position weight matrix of the most occurring 7-mers was plotted using R packages (48).

### Reporter assay

Reporter assays were carried out using constructs of FLAG-NQO1 expressed from the pCMV promoter and driven by either *NQO1* PAS or control *SV40* PAS. Reporter expression levels were analysed by western blot using anti FLAG antibody, and qRT-PCR using a forward primer from FLAG and a reverse primer within the *NQO1* coding sequence, and corresponding *NQO1* cleavage primers as listed in supplementary information.

## RESULTS

### Star-PAP regulates distinct mRNA targets independent of canonical PAPα

**Figure 1.**
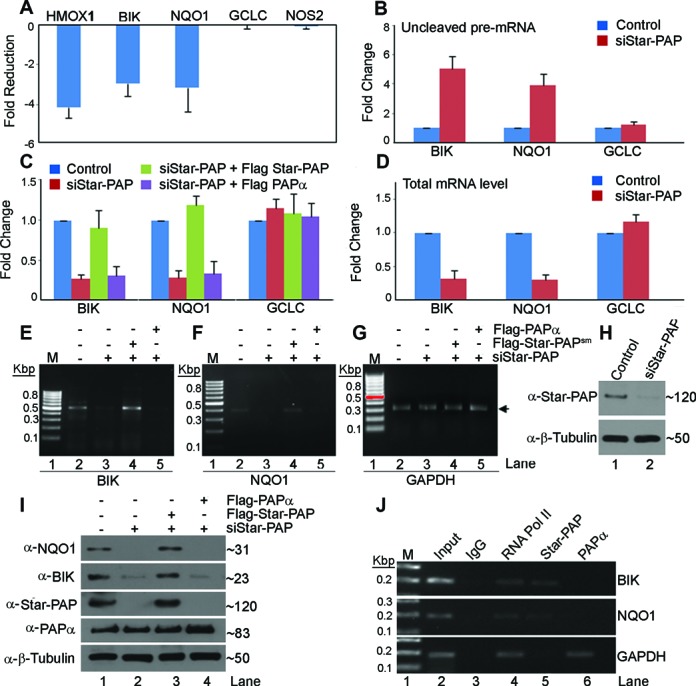
Star-PAP controls distinct mRNA target independent of PAPα. (**A**) qRT-PCR analysis of mRNAs after knockdown of Star-PAP in HEK 293 cells expressed as fold-reductions relative to the control cells. (**B**) Measurement of uncleaved pre-mRNA of *BIK, NQO1* and *GCLC* by qRT-PCR expressed relative to total mRNA level in presence and absence of Star-PAP knockdown. Total mRNA levels are shown in D. (**C**) qRT-PCR analysis of the expression of *BIK* and *NQO1* mRNA level after Star-PAP knockdown followed by rescue with stable expressed FLAG-Star-PAP insensitive to the siRNA used for the knockdown or stable expressed FLAG-PAPα in HEK 293 cells. (**D**) Total mRNA levels of corresponding genes in B. (**E**–**G**) 3′-RACE assay of *BIK, NQO1* and *GAPDH* under the similar conditions as in C. (**H**) Western blot showing knockdown of Star-PAP. (**I**) Western blot of NQO1, BIK and control β-Tubulin from HEK 293 cell lysates after knockdown of Star-PAP and rescue with FLAG-Star-PAP or -PAPα as described in C. Wherever (–) siRNA is indicated, we have used control scrambled siRNA. (**J**) RNA immunoprecipitation analysis of RNA Pol II, Star-PAP and PAPα on UTR RNAs as indicated.

Earlier, it was reported that Star-PAP regulates specific mRNAs and assembles a distinct 3′-end processing complex (40,41). Star-PAP was not detected in the PAPα complex and vice versa. We used bona fide Star-PAP targets *Bcl2 interacting killer, BIK* and *NAD(P)H quinone oxidoreductase 1, NQO1* (41,42) as examples to study the mechanistic difference from PAPα. To confirm the specificity of Star-PAP for target mRNA regulation, Star-PAP was knocked down (Figure 1H) and rescued with stable expressed FLAG-Star-PAP (having silent mutation that renders it insensitive to the siRNA used for Star-PAP knockdown, Star-PAP^sm^)(42) or -PAPα in HEK 293 cells and analysed the expression and 3′-end processing of target pre-mRNAs. As expected there was a loss of *HMOX1, BIK* and *NQO1* mRNA but not *GCLC* or *NOS2* on Star-PAP knockdown (Figure 1A). Loss of *BIK* or *NQO1* mRNA expression was specifically rescued by stable expressions of FLAG-Star-PAP but not by stable expression of FLAG-PAPα (Figure 1C). Western blot analysis corroborated the observation that PAPα does not rescue the loss of BIK or NQO1 protein level due to Star-PAP knockdown (Figure 1I) suggesting specificity of Star-PAP target mRNAs. Measurement of uncleaved pre-mRNA using a pair of primers across the Star-PAP regulated cleavage site (41) on *NQO1* UTR and *BIK* UTR also demonstrated an increased accumulation of uncleaved pre-mRNA on Star-PAP knockdown (Figure 1B, D). Similar results were observed in 3′-RACE assays where the loss of the RACE product of *BIK/NQO1* from Star-PAP knockdown was rescued by stable expression of Star-PAP but not with PAPα (Figure 1E-G), confirming that the 3′-end formation and expression of Star-PAP target mRNAs does not require PAPα, and that it is exclusively controlled by Star-PAP. RNA immunoprecipitation (RIP) experiment demonstrated specific association of Star-PAP with *BIK* or *NQO1* UTR RNA *in vivo* but not with non-target *GAPDH*, and vice versa for PAPα (Figure 1J). Together, these results demonstrate that Star-PAP and PAPα controls distinct mRNA targets.

### Star-PAP competes with PAPα for CPSF binding

**Figure 2.**
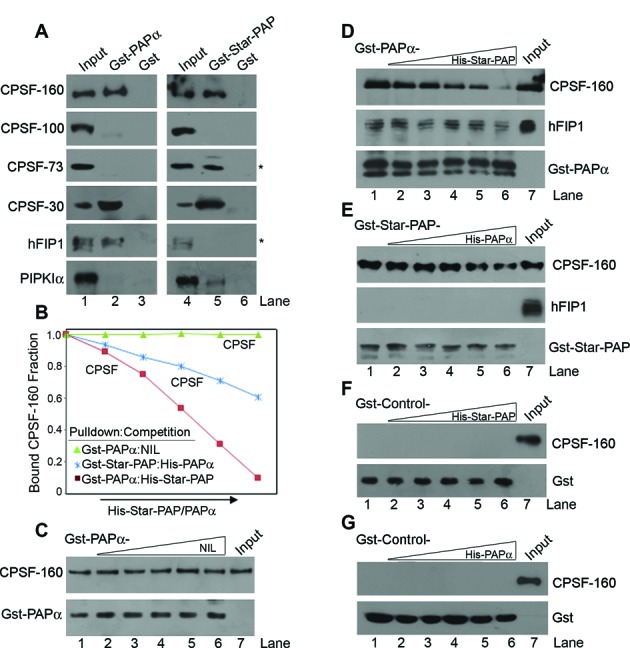
Star-PAP and PAPα compete with each other for CPSF-160 binding. (**A**) GST-pulldown using GST-Star-PAP or -PAPα to pulldown various CPSF subunits from HEK 293 cell lysates. (**B**) Quantifications of gels from C–E, and plot of relative bound CPSF-160 fraction on addition of increasing His-Star-PAP or -PAPα to compete the binding of CPSF with other PAP. (**C**) Control GST-pulldown experiment of CPSF-160 by GST-PAPα with no competitor PAP addition. (**D**) GST-pulldown of CPSF-160 and hFIP1 by GST-PAPα in the presence of increasing additions of (0, 12.5 nM, 25 nM, 50 nM, 100 nM, 200 nM) His-Star-PAP. (**E**) GST-pulldown of CPSF-160 and hFIP1 by GST-Star-PAP in the presence of increasing additions of (0, 12.5 nM, 25 nM, 50 nM, 100 nM, 200 nM) His-PAPα. (**F**–**G**) GST-pulldown experiment with control GST- from HEK 293 cell lysates in the presence of increasing His-Star-PAP and -PAPα additions.

Star-PAP assembles a distinct cleavage complex that contains unique components such as CKIα, PIPKIα and PKCδ (41–43). Previously, Star-PAP association with cleavage factors was shown by immunoprecipitation experiments (41). To compare Star-PAP and PAPα close interactions with various CPSF subunits, we carried out GST-pulldown experiments using recombinant GST-Star-PAP or -PAPα from HEK 293 cell lysates. While GST-Star-PAP pulled down CPSF-160, -73 and -30 kilodalton subunits, GST-PAPα was bound to CPSF-160, -30 kilodalton subunits and hFIP1 but not to CPSF-73 (Figure 2A) suggesting that the two PAPs have different affinities for cleavage factors. Star-PAP co-regulator PIPKIα was specifically detected with Star-PAP but not with PAPα (Figure 2A). CPSF-160 was pulled down by both GST-Star-PAP and -PAPα (Figure 2A). Therefore, to test if the two PAPs compete for CPSF-160 binding, we carried out GST-pulldown experiment using GST-Star-PAP from HEK 293 cell lysates in presence of increasing His-PAPα and vice versa. We observed a decrease in the bound CPSF-160 to Star-PAP on increasing addition of recombinant His-PAPα (Figure 2E). There was no interaction of CPSF-160 with GST- controls in the presence of His-Star-PAP or His-PAPα additions (Figure 2F,G). Similarly, there was subsequent loss of CPSF-160 bound to PAPα in presence of increasing His-Star-PAP addition (Figure 2D), indicating that both PAPs compete with each other for CPSF-160 binding. However, the loss of CPSF-160 bound to PAPα was higher when competed by Star-PAP than the loss of CPSF-160 bound to Star-PAP when competed with PAPα (Figure 2B, D, E). At the maximum concentration His-Star-PAP (200 nM) used for competition, only ∼20% of CPSF-160 remained bound to GST-PAPα, while >50% of CPSF-160 remained bound to GST-Star-PAP when competed with similar concentration of His-PAPα (Figure 2B). These experiments indicate a preferential binding of CPSF-160 to Star-PAP over PAPα. We also tested competition of the two PAPs for another PAP positioning factor, hFIP1 binding. hFIP1 did not significantly associate with Star-PAP, nor any visible competition by Star-PAP for hFIP1 binding to PAPα was observed (Figure 2D, E).

### Sequence motifs around the Star-PAP target UTRs/poly (A) site determines specificity

Previous studies demonstrated Star-PAP binding to target pre-mRNA (40). An enrichment of GC-rich sequence upstream and a deplete U-sequence downstream of poly (A) site on Star-PAP target mRNAs genome wide was reported (42). Moreover, a large footprint of ∼60 nucleotides was observed on target *HMOX1* and *BIK* UTR but no discrete motif was identified (40,42) (Supplementary Figure S1A). A recent ‘RNA compete’ analysis indicated a putative -AUA- enriched motif for Star-PAP binding *in vitro* (45). Moreover, a lack of discernible U/GU-rich DSE for CstF-64 recognition was observed on *BIK* UTR (42) or *NQO1* UTR (Figure 4A, Supplementary Figures S1A and S2A). Other Star-PAP targets also showed similar -AUA- motif around the putative Star-PAP binding site, and the suboptimal DSE with no discernible U/GU-rich sequence (data not shown). These features were not observed on *GCLC* or other Star-PAP non-target UTRs (Supplementary Figure S1A). These sequence elements are likely to play key role in Star-PAP specificity and to keep PAPα out of Star-PAP target mRNAs.

**Figure 3.**
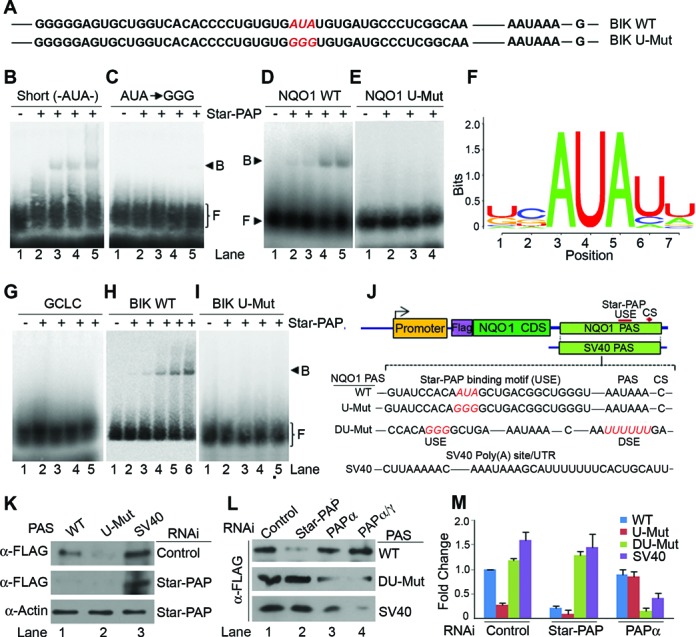
Star-PAP recognition of target mRNA is driven by a -AUA- core motif upstream of PAS. (**A**) *BIK* UTR RNA sequence showing Star-PAP binding region and mutations of AUA motif. (**B**) RNA EMSA experiment of Star-PAP with short RNA oligo having AUA in the sequence (**C**) with AUA to GGG mutation on the oligo (**D**) with *NQO1* UTR RNA (**E**) with a mutation of AUA to GGG on *NQO1* UTR. (**F**) Putative Star-PAP binding motif obtained by *in silico* analysis of Star-PAP target mRNAs at the Star-PAP binding region with core -AUA- motif. (**G**) RNA EMSA experiment of Star-PAP with *GCLC* UTR RNA (**H**) *BIK* UTR and (**I**) *BIK* UTR with AUA to GGG mutation in the Star-PAP binding region. F: free probe, B: Star-PAP-RNA binary complex. (**J**) Schematic of reporter mini gene construct of FLAG-NQO1 expressed from pCMV promoter and driven by *NQO1* PAS (Star-PAP regulated distal poly(A) site that controls overall NQO1 expression, see Supplementary Figure S3) or control *SV40* UTR. The sequence of the Star-PAP binding USE and the mutation of the AUA (U Mut) or introduction of U-rich DSE (D-Mut), or both (DU-Mut) is indicated. (**K** and **L**) Western blot analysis of FLAG-NQO1 HEK 293 cell lysates after transfection of the reporter constructs under the conditions as indicated. (**M**) qRT-PCR analysis of FLAG-NQO1 expression with a forward primer from FLAG and reverse primer from NQO1 CDS from HEK 293 cells after transfection of the reporter constructs.

### Star-PAP recognises a unique nucleotide motif on its target mRNA

To identify the exact Star-PAP recognition motif, we employed a short RNA oligo having AUA (45) and confirmed Star-PAP binding by an *in vitro* EMSA experiment (Figure 3B) using recombinant His-Star-PAP (Supplementary Figure S4H). Changing the AUA to GGG abolished Star-PAP binding to the short oligo (Figure 3C). We recently reported an *in vitro* template for single RNA molecule studies that mimic Star-PAP dependent cleaved UTR having AAUAAA signal followed by a short A stretch and an -AUA- motif upstream of the PAS signal that showed Star-PAP binding as well as Star-PAP dependent polyadenylation (submitted elsewhere). To further study the significance of -AUA- motif in the Star-PAP target mRNA binding, we used an *in vitro* transcribed *BIK* UTR RNA encompassing the Star-PAP footprint region or an equivalent region from *NQO1* UTR and mutated the -AUA- motif to GGG (Figure 3A, Supplementary Figure S2A). EMSA experiments demonstrated Star-PAP specific binding to both *BIK* (Figure 3H) and *NQO1* UTR (Figure 3D) but not to the non-target *GCLC* UTR RNA (Figure 3G). Antibody supershift and competitions with excess of non-radiolabelled specific and non-specific RNA demonstrated the specificity of Star-PAP-*BIK* or -*NQO1* interaction (Supplementary Figure S1B-D). Mutation of AUA to GGG on *BIK* or *NQO1* UTRs abolished Star-PAP binding (Figure 3E, I) indicating that -AUA- motif is required for Star-PAP mRNA binding.

To further study the physiological significance of the -AUA- motif on Star-PAP regulation of target mRNAs, we used a reporter mini gene construct where FLAG-NQO1 under CMV promoter was driven by *NQO1* UTR or control *SV40* UTR (49) (Figure 3J, Supplementary Figure S3D). *NQO1* encodes three mRNA isoforms corresponding to three poly (A) sites at the 3′-UTR (50) (Supplementary Figure S3A), of which the distal poly (A) site was the predominant site that accounts for most NQO1 protein expression (Supplementary Figure S3B). Star-PAP specifically regulates the distal poly (A) site on *NQO1* UTR (Supplementary Figure S3C). In our reporter assay, the expression of FLAG NQO1 driven by distal poly (A) site was indistinguishable from that of full-length *NQO1* UTR having all the three sites (Supplementary Figure S3B, C and E). Thus, Star-PAP controls overall NQO1 expression through regulation of the distal specific poly (A) site (Supplementary Figure S3C, Figure 1I). Therefore, in our reporter assays we employed the *NQO1* distal poly (A) site to define Star-PAP specificity (we refer this as *NQO1* PAS in the paper) (Supplementary Figure S3D). Corresponding mutations were made from -AUA- to -GGG- on the *NQO1* PAS at the Star-PAP binding region (we refer it as upstream mutation, U-Mut) (Figure 3J). The reporter constructs were transfected into HEK 293 cells and the expression of FLAG-NQO1 was measured by Western blot using anti-FLAG antibody and qRT-PCR using a forward primer at the FLAG sequence and reverse primer in the *NQO1* CDS. We confirmed the Star-PAP dependent expression of FLAG-NQO1 reporter construct driven by *NQO1* PAS by 3′-RACE assays, western and qRT-PCR after Star-PAP knockdown in HEK 293 cells (Supplementary Figure S3G-I). The reporter assay showed loss of FLAG-NQO1 expression driven by *NQO1* PAS but not by *SV40* upon Star-PAP knockdown (Figure 3K-M, Supplementary Figure S3G-I). Given that *SV40* is a more robust poly (A) site than *NQO1, SV40* UTR driven FLAG-NQO1 showed higher protein expression than that of *NQO1* PAS (Figure 3K, Supplementary Figure S3B). Consistent with the EMSA results, mutation of AUA to GGG in the reporter construct driven by *NQO1* PAS resulted in decreased FLAG-NQO1 expression both in protein (Figure 3K) and RNA levels (Figure 3M). These results phenocopy Star-PAP knockdown (Figure 3K, Supplementary Figure S3G-I) demonstrating that -AUA- motif is critical for Star-PAP mediated NQO1 regulation. It also validated our earlier result that *NQO1* PAS is an exclusive Star-PAP target and loss of Star-PAP regulation diminished its expression. PAPα, however, was unable to access *NQO1* PAS even in the absence of Star-PAP (Figure 3L, M) indicating that Star-PAP binding is not the reason for PAPα exclusion from the target UTR. However, PAPα knockdown had a modest effect on *SV40* PAS driven FLAG-NQO1 expression that was diminished by knockdown of PAPα and its close paralog PAPγ (Figure 3L). *NQO1* PAS driven reporter expression was not affected by either the individual knockdowns of PAPα and PAPγ, or double knockdowns of both the canonical PAPs (Figure 3L, M, Supplementary Figure S3E). This also confirms that *NQO1* PAS is not regulated by canonical PAPs, PAPα or PAPγ, and is exclusive to Star-PAP. Interestingly, mutation of AUA to GGG when combined with an insertion of a U-rich DSE (UUUUUU at the DSE) (DU-Mut, described in sections below) rendered the *NQO1* PAS driven reporter expression independent of Star-PAP regulation, and was specifically controlled by PAPα (and not by PAPγ) (Figure 3L, M, Supplementary Figure S3E). These results confirm that Star-PAP regulation requires -AUA- motif that acts as a core Star-PAP recognition sequence.

We then analysed *in silico* for all the genes down-regulated by Star-PAP knockdown from earlier microarray data (41) at the corresponding Star-PAP binding regions as on *BIK* or *HMOX1* (40,42) (we used −50 to −150 nucleotides upstream of polyA site) for the presence of -AUA- motif (Supplementary Table S1). We observed the occurrence of -AUA- motif in >80% of Star-PAP regulated genes (Supplementary Figure S5A, Table S1). A control data set of randomly selected Star-PAP non-regulated genes showed -AUA- present in <50% of the genes in the corresponding region (Supplementary Figure S5B). This indicates a prevalence of -AUA- containing motif among the Star-PAP target genes in the specified region. Interestingly, -AUA- motif was detected mostly between −60 and −120 upstream of poly (A) site consistent with earlier footprints (Supplementary Figure S5A). We then looked for the most commonly occurring 5-mers having AUA, and showed ∼12 sequences (from 48 possible combinations of 5-mers) that were more frequently observed than the rest (occurs in >55% of the genes) (Supplementary Figure S5A). We then identified 7-mers containing the above-mentioned most frequently occurring 5-mers with AUA, and keeping the AUA element at the central position, we obtained a putative Star-PAP recognition motif (with enriched –AUAU– among the identified 5- and 7-mers) (Figure 3F, Supplementary Figure S5D). The list of 5-mers and 7-mers sequences detected from the Star-PAP target genes are shown in Supplementary Table S2. This region on Star-PAP target genes were earlier reported to have enriched G and C nucleotides compared to the non-target genes (42). Therefore, AUA motif along with the GC-rich region around it could serve as Star-PAP recognition sequence and endow specificity for its target poly (A) site (s).

### Suboptimal DSE prevents CstF-64 binding to Star-PAP target mRNAs

**Figure 4.**
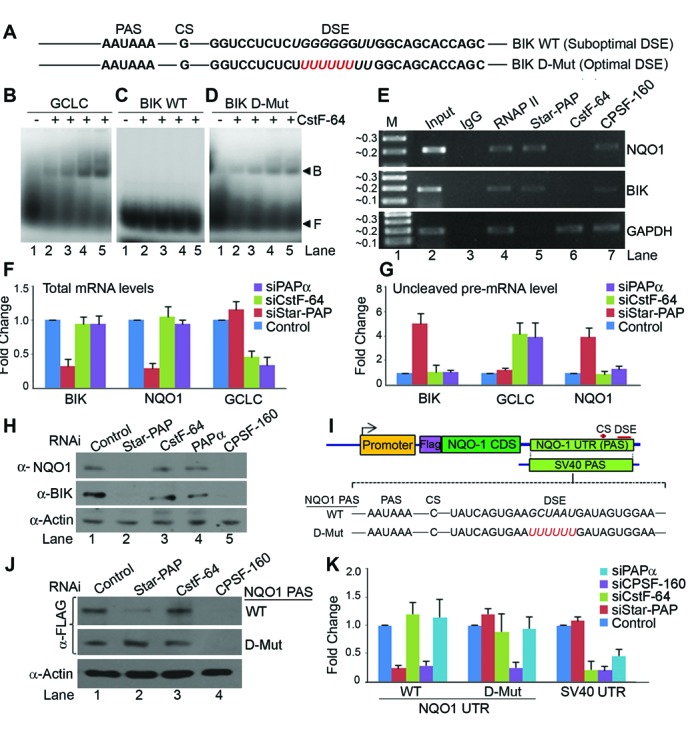
Suboptimal DSE at the 3′-UTR of Star-PAP target mRNAs prevent CstF-64 binding that excludes PAPα from the UTR. (**A**) *BIK* UTR sequence indicating the suboptimal DSE region and the insertion of U-rich DSE (UUUUUU) at the UTR. RNA EMSA experiment of CstF-64 with (**B**) *GCLC* (**C**) *BIK* UTR and (**D**) mutant *BIK* with U-rich DSE. F-free probe, B- CstF-64-RNA binary complex. (**E**) RIP analysis of Star-PAP, CstF-64, CPSF-160 and RNA Pol II on mRNAs as indicated. (**F**) qRT-PCR analysis of *BIK, NQO1* and *GCLC* mRNA expressions under the conditions as indicated. (**G**) Uncleaved mRNA measurement by qRT-PCR expressed relative to the total mRNA levels under conditions as in F. (**H**) Western blot analysis of NQO1, BIK and control α-Actin from lysates of HEK 293 cells after knockdown of Star-PAP, CstF-64, CPSF-160, PAPα or control cells. (**I**) Reporter constructs as in Figure 3J, showing DSE region and the U-rich DSE insertions. (**J**) Reporter assay by western blot analysis using anti-FLAG antibody from HEK 293 cells after transfection of the reporter construct under the conditions as indicated. (**K**) qRT-PCR analysis under the similar conditions as in J.

Star-PAP target mRNAs are independent of PAPα. Yet it is obscure how PAPα is unable to process Star-PAP target pre-mRNA 3′-ends despite the presence of canonical AAUAAA signal at the 3′-UTR. In the case of *BIK* or *NQO1*, DSE is suboptimal with no discernible U/GU-rich sequence for CstF-64 recognition (Figure 4A, Supplementary Figure S1A, S2A). To test the role of U-deficit DSE, we used *in vitro* transcribed short UTR RNA fragment from *BIK* and *NQO1* PAS encompassing the downstream UTR (DSE) region (Figure 4A, Supplementary Figure S2A) in EMSA experiments with recombinant His-CstF-64 (Supplementary Figure S4I). Since CstF-64 binding is generally weak, we UV-crosslinked the UTR RNA with His-CstF-64 in solution before resolving on the gel. While CstF-64 was bound to control *GCLC* UTR (Figure 4B), it did not show significant binding to *BIK* (Figure 4C) or *NQO1* UTR (Supplementary Figure S2C). We then introduced a U-rich sequence (UUUUUU) at the DSE on *BIK* and *NQO1* UTR to make it proficient for CstF-64 interaction (4,33) (Figure 4A, Supplementary Figure S2A). Concomitantly, introduction of the U-rich DSE resulted in significant CstF-64 binding to *BIK* UTR RNA in similar EMSA experiments (Figure 4D). Similar results were obtained for *NQO1* UTR RNA as well (Supplementary Figure S2D), illustrating that suboptimal DSE prevented CstF-64 binding to the Star-PAP target mRNA UTRs. Competitions with specific and non-specific RNA fragments showed specificity of the CstF-64 interaction with *GCLC, BIK* or *NQO1* UTR RNA (Supplementary Figure S2B, E, F). Further, we tested the association of CstF-64 with *BIK* or *NQO1* UTR RNA by RIP analysis in HEK 293 cells. Interestingly, while CPSF-160 was equally crosslinked with *BIK, NQO1* and *GAPDH* UTR RNAs, CstF-64 was not detected on *BIK* or *NQO1* UTR (Figure 4E), confirming our observations from EMSA experiment. Star-PAP and RNAP II were detected on both *BIK* and *NQO1* UTRs (Figure 4E). These results indicate that CstF-64 does not bind Star-PAP target mRNA UTRs and is likely dispensable for Star-PAP dependent 3′-end processing.

### CstF-64 is dispensable for the expression/3′-end processing of Star-PAP target mRNAs

To explore the role of CstF-64 on the expression and 3′-end processing of Star-PAP target mRNAs, we knocked down CstF-64 (51) (Supplementary Figure S4D) in HEK 293 cells and analysed the expression profiles of *BIK* and *NQO1* mRNA. Loss of CstF-64 did not affect the mRNA expression levels of *BIK* or *NQO1*, while it reduced the levels of *GCLC* mRNA (Figure 4F) indicating that CstF-64 is not required for the processing of Star-PAP target mRNAs. Western blot analysis also showed loss of both BIK and NQO1 protein expressions (Figure 4H) upon Star-PAP (Supplementary Figure S4A) or CPSF-160 knockdown (52) (Supplementary Figure S4C), but no effect on CstF-64 knockdown (Figure 4H). The loading control actin/tubulin was not affected by the knockdowns of CPSF-160, CstF-64, PAPα or other cleavage factors tested (Supplementary Figure S4A-G) consistent with earlier studies (52–58). We then analysed the 3′-end processing of *BIK/NQO1* by measuring the uncleaved pre-mRNA or 3′-RACE assay after CstF-64 or Star-PAP knockdowns. There was increased accumulation of *BIK* or *NQO1* uncleaved pre-mRNA on Star-PAP knockdown; however CstF-64 knockdown did not affect the cleavage efficiency of *BIK* or *NQO1* UTR (Figure 4G). We observed similar results with 3′-RACE assay where CstF-64 knockdown did not show any effect on mature poly (A) tailed mRNA synthesis of *BIK* or *NQO1* (data not shown). We also showed that *NQO1* PAS or its respective mutants were independent of CstF-64τ, another close paralog of CstF-64 (Supplementary Figure S3F, K). Together, these results confirm that CstF-64 is dispensable for the expression and 3′-end processing of Star-PAP target RNAs. CstF-64 binding to the DSE is critical for the assembly of CPSF complex at the PAS and recruitment of PAPα (4,33). Our results suggest that the lack of CstF-64 binding to Star-PAP target mRNAs is likely to render PAPα inaccessible for recruitment and thus excluding PAPα from Star-PAP target pre-mRNAs.

### Lack of CstF-64 binding excludes PAPα from Star-PAP target pre-mRNA UTRs

To further confirm the role of CstF-64 and suboptimal DSE sequence on the expression and 3′-end processing Star-PAP target mRNAs, we used the reporter FLAG-NQO1 mini gene construct described in the earlier section. We introduced a U-rich DSE (UUUUUU) to make it proficient for CstF-64 recognition (we refer it as downstream mutant, D-Mut) (Figure 4I). After transfection of the reporter construct, FLAG-NQO1 expression was measured by Western blot and qRT-PCR as described in previous section. Knockdown of Star-PAP resulted in the loss of reporter FLAG-NQO1 expression from *NQO1* PAS in both Western and qRT-PCR analysis (Figure 4J, K). Consistently, CstF-64 or CstF-64τ knockdown did not show any effect on the FLAG-NQO1 levels driven by wild type *NQO1* PAS (Supplementary Figure S3F). Knockdown of both CstF-64 and CstF-64τ together also did not affect the *NQO1* PAS regulated expression (data not shown). Both *SV40* and *NQO1* UTR driven reporter expressions were diminished by CPSF-160 knockdown (Figure 4J, K). Strikingly, introduction of U-rich sequence at the DSE resulted in the loss of Star-PAP exclusive control of *NQO1* PAS driven reporter. Star-PAP knockdown no longer affected the FLAG-NQO1 expression (Figure 4J, K) suggesting that *NQO1* PAS driven construct can be regulated by PAPα in presence of U-rich DSE. Yet, CstF-64 knockdown still had no effect on the FLAG-NQO1 expression as Star-PAP could process the UTR in absence of CstF-64 or PAPα. Consistently, knockdown of either PAPα (Supplementary Figure S4B) or Star-PAP did not affect the expression of FLAG-NQO1 in the presence of U-rich DSE (Figure 4K) indicating that the FLAG-NQO1 reporter construct was regulated redundantly by both Star-PAP and PAPα. Taken together these results indicate that it is the lack of CstF-64 binding that prevented PAPα in accessing Star-PAP target mRNAs due to its suboptimal DSE.

### Mutation of AUA at the USE and introduction of U-rich DSE converts a Star-PAP regulated mRNA into a canonical PAPα target

We have shown that introduction of U-rich DSE at the *NQO1* PAS results in the loss of Star-PAP exclusive control over *NQO1* expression and it becomes target for both the PAPs (Figure 4J, K). However, when the Star-PAP binding motif was mutated from AUA to GGG and in presence of U-rich DSE at the *NQO1* PAS (DU-Mut), FLAG-NQO1 reporter expression was no longer controlled by Star-PAP (Figure 3L, M). Knockdown of Star-PAP did not affect protein or mRNA expression levels of DU-Mut *NQO1* PAS driven FLAG-NQO1 reporter (Figure 3L-M). Instead, there was loss of expression of FLAG-NQO1 upon PAPα knockdown (Figure 3L-M). Since PAPγ is mechanistically and functionally similar to PAPα, we tested if FLAG-NQO1 expression was also dependent on PAPγ. Knockdown of either PAPγ or control Star-PAP did not have any effect on the expression of FLAG-NQO1 from the DU-Mut reporter construct (Supplementary Figure S3E). Moreover, exogenous expression of PAPα but not PAPγ or Star-PAP rescued the loss of FLAG-NQO1 expression on PAPα knockdown (Supplementary Figure S3J), suggesting that the DU-Mut driven FLAG-NQO1 reporter expression was controlled by PAPα. Similarly, knockdown of CstF-64 but not CstF-64τ diminished the DU-Mut driven FLAG-NQO1 reporter expression (Supplementary Figure S3F) suggesting the specific involvement of CstF-64 in the regulation. Whereas the wild-type (WT) *NQO-1* PAS driven reporter was affected neither by PAPα/PAPγ nor by CstF-64/CstF-64τ knockdown, it was specifically controlled by Star-PAP (Supplementary Figure S3E, F). 3′-RACE assay also confirmed the loss of Star-PAP regulation and switch over to PAPα as its regulator PAP (data not shown). Control CPSF-160 knockdown resulted in the loss of expression of *NQO1* PAS driven reporter both in the presence and absence of the U-rich DSE insertions. Thus, altering the DSE in presence of -AUA- motif mutation on Star-PAP target mRNA UTR, switches the regulating PAP from Star-PAP to PAPα.

## DISCUSSION

**Figure 5.**
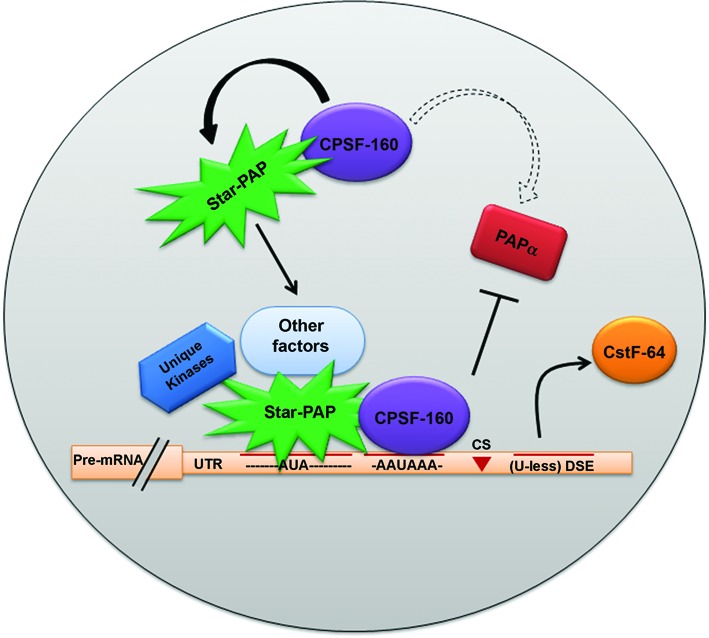
Model of Star-PAP mediated poly(A) site/UTR selection. Star-PAP recognition of AUA motif and suboptimal DSE mediated exclusion of PAPα is indicated.

Star-PAP is a non-canonical nuclear PAP that selects pre-mRNA targets for polyadenylation (40,41). Studies on Star-PAP demonstrated specificity of PAPs for mRNA UTR/poly (A) site selection, yet the mechanism of PAP specificity remains elusive. Our results illustrated the specificity elements of Star-PAP mediated UTR/poly (A) site selection that excludes PAPα. A model of Star-PAP target specificity is depicted in Figure 5. Star-PAP recognition of a core nucleotide AUA element and a suboptimal DSE directs Star-PAP exclusive control over its target mRNAs. Conversely, lack of Star-PAP binding sequence will keep Star-PAP out of the other non-target pre-mRNAs, indicating distinct target poly (A) sites for the two PAPs. Moreover, both PAPs assemble distinct complexes with different interacting partners. Star-PAP is not detected with PAPα and vice versa (41). This supports an earlier proposed model for ‘PAP selection’ at the 3′-end where distinct sequences at the 3′-UTR selects specific PAP for polyadenylation (2). This idea is reinforced by our observation that changing sequence elements at the 3′-UTR on Star-PAP target mRNAs can switch the regulatory PAP from Star-PAP to PAPα. This target UTR specificity/selection will have direct implication on the regulation of alternative polyadenylation (APA) when poly (A) sites regulated by both PAPs are present on a single pre-mRNA 3′-UTR (59,60). Currently, there is no example so far reported of the two PAPs selecting different poly (A) sites on the same pre-mRNA. Similar target mRNA specificities of PAPs is known in plants that modulates growth and pathogen interaction in arabidopsis (61). Difference in the poly (A) site usage pattern of the two mammalian canonical PAPs (PAPα and PAPγ) in APA regulation is also reported (57).

Nevertheless, both PAPs—Star-PAP and PAPα are involved in the basic 3′-end processing reaction—cleavage and polyadenylation although with different mechanisms (2,4,33). The role of PAPα in the cleavage reaction is undefined, while Star-PAP plays a structural role that recruits CPSF-160 and -73 to assemble a stable cleavage complex (40). Interestingly, both PAPs differ in interactions with key cleavage factors while compete with each other for CPSF-160, but with a preference for Star-PAP over PAPα. Given Star-PAP's direct binding to mRNA and the predominant interaction with CPSF-160, it is likely that Star-PAP target poly (A) sites are preferentially cleaved over the canonical poly (A) site (s) thus encoding more mRNA/protein. However, the physiological significance of such preferential binding is yet to be established.

There are two aspects of PAP specificity—recognition of distinct UTR/poly (A) site, and exclusion of the other PAP. How PAPα/γ recognises specific poly (A) site or excludes other PAPs is vague, but is likely through the cleavage factors (4,33). For Star-PAP the first aspect is defined by a specific RNA element on the target UTR. Star-PAP has a large footprint (∼60 nucleotides) with a GC-rich sequence that contains the AUA motif (40,42). Moreover, a general enrichment of GC was reported upstream of PAS on Star-PAP target mRNAs (45). These sequences are likely to contribute to Star-PAP recognition in addition to the core -AUA- motif putatively as accessory or regulatory elements. Star-PAP mRNA association is regulated *in vivo* by PIPKIα or PI4,5P_2_ binding, and/or phosphorylation (42–44). CKIα mediated phosphorylation at the serine 6 (S6) on Star-PAP recognised specific subset of target mRNAs independent of other phosphorylations, indicating mRNA specificity mediated by Star-PAP phosphorylation (49). Thus, these signals may direct recognition of distinct motifs on target pre-mRNAs (2,62). The GC-rich sequence around the -AUA- motif on Star-PAP binding region is likely to be critical for such regulations.

The second aspect of Star-PAP specificity is driven by the suboptimal downstream (DSE) sequence that prevents CstF-64 binding. This demonstrates the significance of sequences around the PAS in determining the PAP to be recruited at the 3′-end. In the canonical pathway, CPSF that recognises AAUAAA signal (the actual subunit that recognises PAS is controversial)(11,15–16,18) co-operates with CstF and assembles a stable cleavage complex along with other cleavage factors, symplekin, PABPN1 and PAPα which is recruited to the complex (4,33). Star-PAP in contrast binds the pre-mRNA, recruits CPSF-160 and helps assemble a cleavage complex (40). Star-PAP, CPSF-160 and CPSF-73 reconstitutes cleavage reaction of its target *HMOX1* UTR RNA *in vitro*, suggesting that limited cleavage factors are required for Star-PAP mediated 3′-end processing. Our study confirmed that CstF-64 is dispensable for the processing of Star-PAP target mRNAs. The direct binding of Star-PAP to target mRNA may likely bypass the requirement of positioning factors such as hFIP1 or other cleavage factors (11–12,18). Co-effectors, PIPKIα/CKIα have regulatory role in Star-PAP mediated cleavage and polyadenylation (41–43). Our results show PAP specificity for target poly (A) sites mediated through sequence elements that has potential implications on APA regulation.

## Supplementary Material

SUPPLEMENTARY DATA
