# Supplementary material for: CstF-64 and 3′-UTR cis-element determine Star-PAP specificity for target mRNA selection by excluding PAPα
Source: Nucleic Acids Res. 2015 Oct 22;44(2):811–23. doi: 10.1093/nar/gkv1074 (PMC4737136; doi:10.1093/nar/gkv1074)
Supplement: SUPPLEMENTARY DATA [file supp_44_2_811__index.html]

CstF-64 and 3′-UTR cis-element determine Star-PAP specificity for target mRNA selection by excluding PAPα — SUPPLEMENTARY DATA 

# CstF-64 and 3′-UTR *cis*-element determine Star-PAP specificity for target mRNA selection by excluding PAPα

## SUPPLEMENTARY DATA

- SUPPLEMENTARY DATA
- SUPPLEMENTARY DATA
- SUPPLEMENTARY DATA
