# Supplementary material for: CstF-64 and 3′-UTR cis-element determine Star-PAP specificity for target mRNA selection by excluding PAPα
Source: Nucleic Acids Res. 2015 Oct 22;44(2):811–23. doi: 10.1093/nar/gkv1074 (PMC4737136; doi:10.1093/nar/gkv1074)
Supplement: SUPPLEMENTARY DATA [file supp_gkv1074_nar-01841-a-2015-File007.pdf]

Supplementary Information

Title

CstF-64 and 3'UTR *cis*-element determine Star-PAP specificity for target mRNA selection by excluding PAPα

Divya T. Kandala, Nimmy Mohan, Vivekanand A, Sudheesh AP, Reshmi G, and Rakesh S. Laishram#

Cancer Research Program, Rajiv Gandhi Centre for Biotechnology, Trivandrum - 014, India

Corresponding Author

Running Title: Specificity mechanisms of nuclear poly(A) polymerase Star-PAP

#Corresponding Address:

Rakesh S. Laishram

Rajiv Gandhi Centre for Biotechnology

Thycaud Post, Poojappura

Trivandrum – 695014, India

E mail: laishram@rgcb.res.in

Phone: +91-0471-2529592

List of Antibodies:

Mouse monoclonal anti-FLAG M2 (Sigma), Rabbit monoclonal anti-FLAG (Sigma), Rabbit polyclonal anti-CPSF1 (Bethyl Lab), Rabbit polyclonal anti-CPSF2 (Bethyl lab), Rabbit polyclonal anti-CPSF3 (Bethyl lab), Rabbit polyclonal anti-CPSF4 (Bethyl lab), Rabbit polyclonal anti-CSTF2 (Bethyl lab), Rabbit Polyclonal anti-CSTF2T (Bethyl lab), Rabbit monoclonal anti-β-Tubulin (Novus), Mouse monoclonal anti-Actin (Novus), Mouse monoclonal anti-Gst tag antibody (Cell Biolabs), Rabbit polyclonal anti-PIPKIα (Mellman et al, 2008), Rabbit polyclonal anti-Star-PAP (Mohan et. al. 2015), Rabbit polyclonal anti-PAPOLA (Bethyl lab), Rabbit Polyclonal anti-PAPOLG (Bethyl lab), Rabbit polyclonal anti-hFIP1 (Bethyl), Mouse monoclonal anti-NQO1 (Novus), Rabbit polyclonal anti-RNA Polymerase II (Bethyl lab), Goat polyclonal anti-BIK (Santa Cruz).

Gene specific forward primers used for 3'-RACE:

1. *HMOX1* 5'-CATCCTCAGTTCCTGCAGCA-3'
2. *NQO1* 5'-AAAACACTGCCCTCTTGTGG-3'
3. *GAPDH* 5'-TTTGGCTACAGCAACAGGGT- 3'
5. *BIK* 5'-GCTGGAACACTGCTGAGGTT-3'

Primers used for 3'-end cleavage:

1. *NQO1* Clv FP: 5'-TGCCTTCATCTTCACTGCAA-3'
   Clv RP: 5'-TTGTCAAGCCAGTCACCAAG-3
2. *GCLC* Clv FP: 5'-ATGCCTGGTTTTCGTTTGCA-3'

Clv RP: 5’-AGCTGTGGAACTCACACACACTCA-3’

3. *GAPDH* Clv FP: 5’-CACACTGAATCTCCCCTCCT-3’
   Clv RP: 5’-TTGACACAAGCCCAGCTTC-3’

4. *BIK* Clv FP: 5’-GCCCTCGGCAAAGAATCTAC- 3’
   Clv RP: 5’-CCTTTTGCAGGGAAGGATCT-3’

Primers for RIP analysis:

1. NQO1 FP: 5'-TGCTCTCGACAGTATCCACAA-3'
   RP: 5'-AGTTGTCAAGCCAGTCACCAA-3'

2. *GAPDH* FP: 5'-CACACTGAATCTCCCCTCCT-3'
   RP: 5'-TTGACACAAGCCCAGCTTC-3'

3. *BIK* FP: 5'-TCTTGATGGAGACCCTCCTG-3'
   RP: 5'-GTCCTCCATAGGGTCCAGGT-3'

4. *SV40* FP: 5’- CCCCCTGAACCTGAAACATA-3’
   RP: 5’- TTGGACAAACCACAACTAGAATG-3’

Primers used in quantitative real time PCR:

1. *HMOX1* FP: 5'-CCACCAAGTTCAAGCAGCTCTA -3'
   RP: 5'-GCTCCTGCAACTCCTCAAAGAG-3'

2. *BIK* FP: 5'-TCTTGATGGAGACCCTCCTG-3'
   RP: 5'-GTCCTCCATAGGGTCCAGGT-3'

3. *GCLC* FP: 5'-AAGTTCTTGAAACTCTGCAAGAGAAGG-3'
   RP: 5'-GCCTCAACTGTATTGAACTCGGAC-3'

4. *GAPDH* FP: 5'-GAAGGTCGGAGTCAACGGATTT-3'
RP: 5'-GAATTTGCCATGGGTGGAAT-3'

5. *NQO1* FP: 5'-GAACTTCAATCCCATCATTTCCAG-3'
RP: 5'-CAGCTTCTTTTGTTCAGCCACAAT-3'

6. *NOS2* FP: 5’-CTCAGCTCATCCGCTATGCT-3’
RP: 5’-TCAGGTGGGATTTCGAAGAG-3’

7. FLAG-NQO1 FP: 5’-ATGGATTACAAGGATGACGACG-3’
RP: 5’-CCTCCTTCATGGCATAGTTGAA-3’

siRNA Oligos used:

1. Control scrambled non-targeting: AGGUAGUGUAAUCGCCUUG
2. Star-PAP: GUGUGUUUGUCAGUGGCUU
3. CSTF2: GUUAGAUGCCAGAGGAUUA
4. CSTF2T: CCAUUAUUGACUCACCCUA
5. CPSF1: On target Smart pool (Dharmacon)
6. PAPα: GGAGACUGACUGCGUACUU
7. PAPγ: GCUGGUGGUUCUUGGUAAA

Supplementary Figure Legend:

Supplementary Figure 1: (A) UTR RNA sequences of various Star-PAP target and non-target mRNAs; Star-PAP binding USE region, -AUA- binding motif, poly(A) signal, cleavage site and downstream sequence element (DSE) are indicated. (B) EMSA experiment of *BIK* UTR RNA

with His-Star-PAP in presence and absence of Star-PAP antibody, (C) EMSA of *BIK* UTR RNA with Star-PAP in presence of 100-fold excess of non-radiolabelled specific (S) and non-specific (N) RNA, (D) Competition experiment of *NQO1* RNA in EMSA experiment as in C. F- free probe, B- Star-PAP-RNA binary complex, T- Star-PAP-RNA-Antibody ternary complex.

Supplementary Figure 2: (A) Schematic of *NQO1* UTR sequence showing Star-PAP binding USE region, suboptimal DSE, and Star-PAP binding and DSE mutations, (B) Specific (S) and non-specific (N) competitions in EMSA of *GCLC* with His-CstF-64 as in Suppl. Fig 1C, (C-D) EMSA of *NQO1* PAS and mutant DSE (U-rich DSE insertion, D-Mut) with increasing His-CstF-64 as in Fig. 4B, (E-F) Specific and non-specific competitions in EMSA of *NQO1* and *BIK* downstream mutant UTR RNA with His-CstF-64 as in B. F- free probe, B- Star-PAP-RNA binary complex.

Supplementary Figure 3: Reporter assay using mini-gene FLAG-NQO1 construct. (A) Schematic of *NQO1* UTR with three poly(A) sites, distal site that controls overall NQO1 expression and regulated by Star-PAP is referred as *NQO1* PAS in this paper.(B) Reporter FLAG-NQO1 expression from the reporter constructs as in Fig. 3J with different poly(A) sites of *NQO1* UTR (proximal, middle, distal, or full length UTR having all three polyA sites). Distinct poly(A) site driven FLAG-NQO1 reporter constructs were made by introducing respective mutations at the AAUAAA poly(A) signals as appropriate on the full length *NQO1* UTR. *SV40* UTR was used as a control in the reporter expression experiments. (C) FLAG-NQO1 expression from various reporter constructs as in B after Star-PAP knockdown. (D) Schematic of FLAG-NQO1 reporter construct driven by Star-PAP regulated distal poly(A) site (*NQO1* PAS) that controls overall NQO1 protein expression, or control reporter with *SV40* UTR construct. (E-F) Reporter assay of FLAG-NQO1 with *NQO1* PAS (WT), Star-PAP independent construct of

NQO1 PAS with AUA mutation and U-rich DSE insertion (DU-Mut), or full-length *NQO1* UTR having all three poly(A) sites (FL) after knockdown of PAPα, PAPγ or Star-PAP, and CstF-64 or CstF-64τ as indicated. Westerns for respective knockdowns as well as loading control β-tubulin is also shown. (G) 3'-RACE assay of FLAG-NQO1 reporter from total RNA isolated from the cell after transfection of the reporter constructs in the presence and absence of Star-PAP knockdown, (H) Reporter assay of FLAG-NQO1 by Western blot using anti-FLAG antibody under the conditions as indicated, (I) Measurement of cleavage efficiency using a pair of primers across the cleavage site of the reporter NQO1 and total mRNA expression using a pair of primer from FLAG and NQO1 reading frame under the conditions indicated. (J-K) Reporter expression of the Star-PAP independent *NQO1* PAS mutant (DU Mut) after PAPα knockdown and rescue experiment with exogenous expression of PAPα, PAPγ and Star-PAP, or after CstF-64 knockdown and rescue experiment with exogenously expressed CstF-64τ. Wherever (–) siRNA is indicated, we have used control scrambled siRNA.

Supplementary Figure 4: Western blots showing knockdown of (A) Star-PAP, (B) PAPα, (C) CPSF-160, (D) CstF-64, (E) PAPγ, and (F) CstF-64τ in HEK 293 cells. (G) Actin protein level after knockdown of several 3’-end processing factors. Knockdown blots are shown in A-F. (H) Coommassie stained gel showing purified His-Star-PAP and (I) His-CstF-64.

Supplementary Figure 5: *In silico* analysis of -AUA- containing sequences genome wide on Star-PAP target genes (A) Line plot of -AUA- motif occurrence among Star-PAP target genes in the putative Star-PAP binding region (from -50 to -150 upstream of polyA site) showing the position of occurrence. The list of genes and position of AUA and their frequency of occurrence is shown in Suppl. Table 1. (B) Similar line plot as in A using a negative set of randomly selected Star-PAP non-regulated genes. (C) 12-most frequently occurring 5-mers (from the total

possible 48 sequence having AUA) that occurred in >55% of the genes, (D) Top 15-most commonly observed 7-mers with central AUA motif and the corresponding p-values. Enrichment of -AUAU- motif is indicated.

Supplementary Table 1: Occurrence of -AUA- along with their position and frequency from Star-PAP regulated genes within the putative Star-PAP binding region (-50 to -150 upstream of polyA site).

Supplementary Table 2: List of 5-mers and 7-mers along with the confidence 7-mer obtained from the most frequently occurring 5-mers along with their frequency of occurrences in the dataset.

Supplementary Figure 1

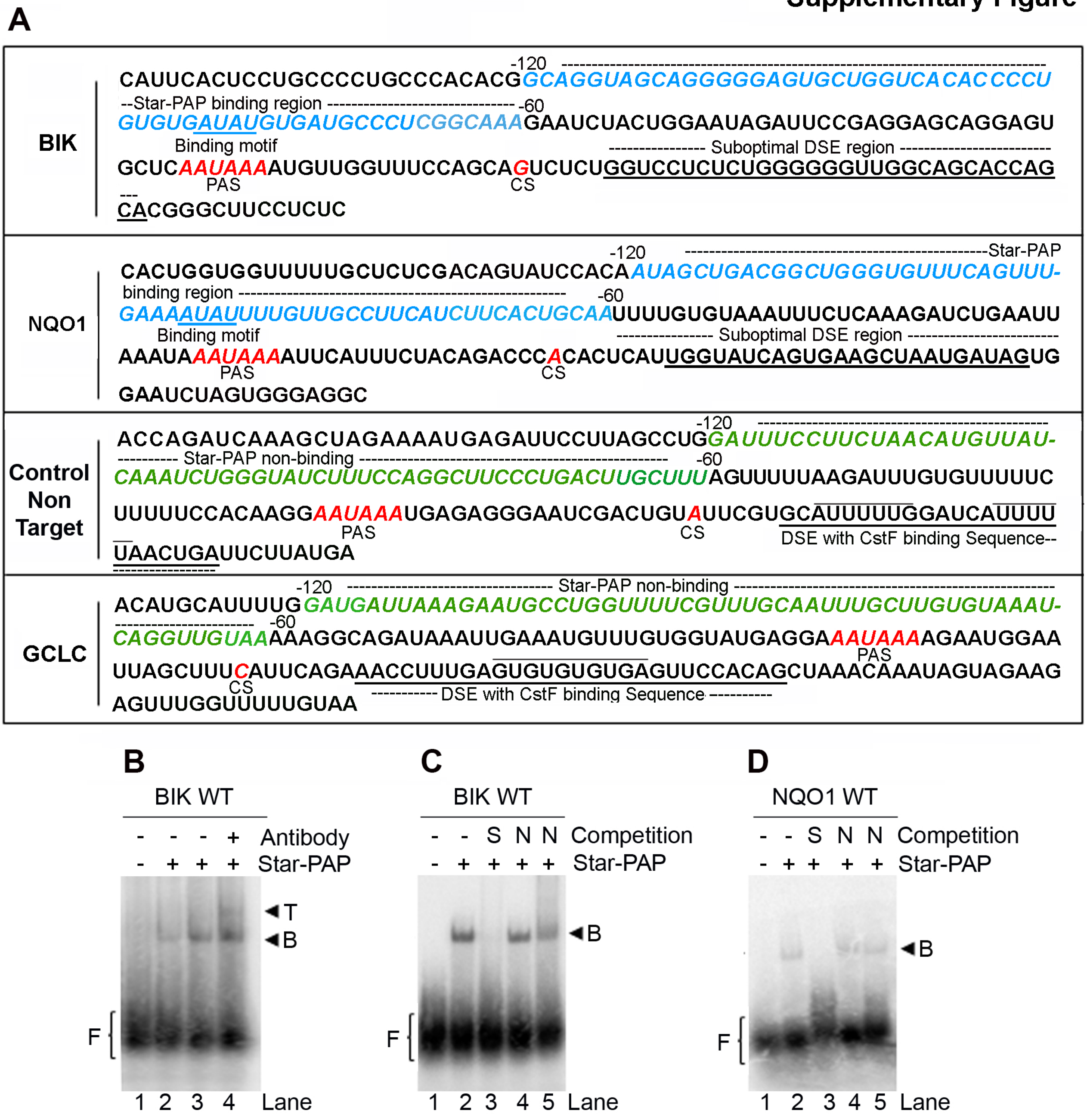

Supplementary Figure 2

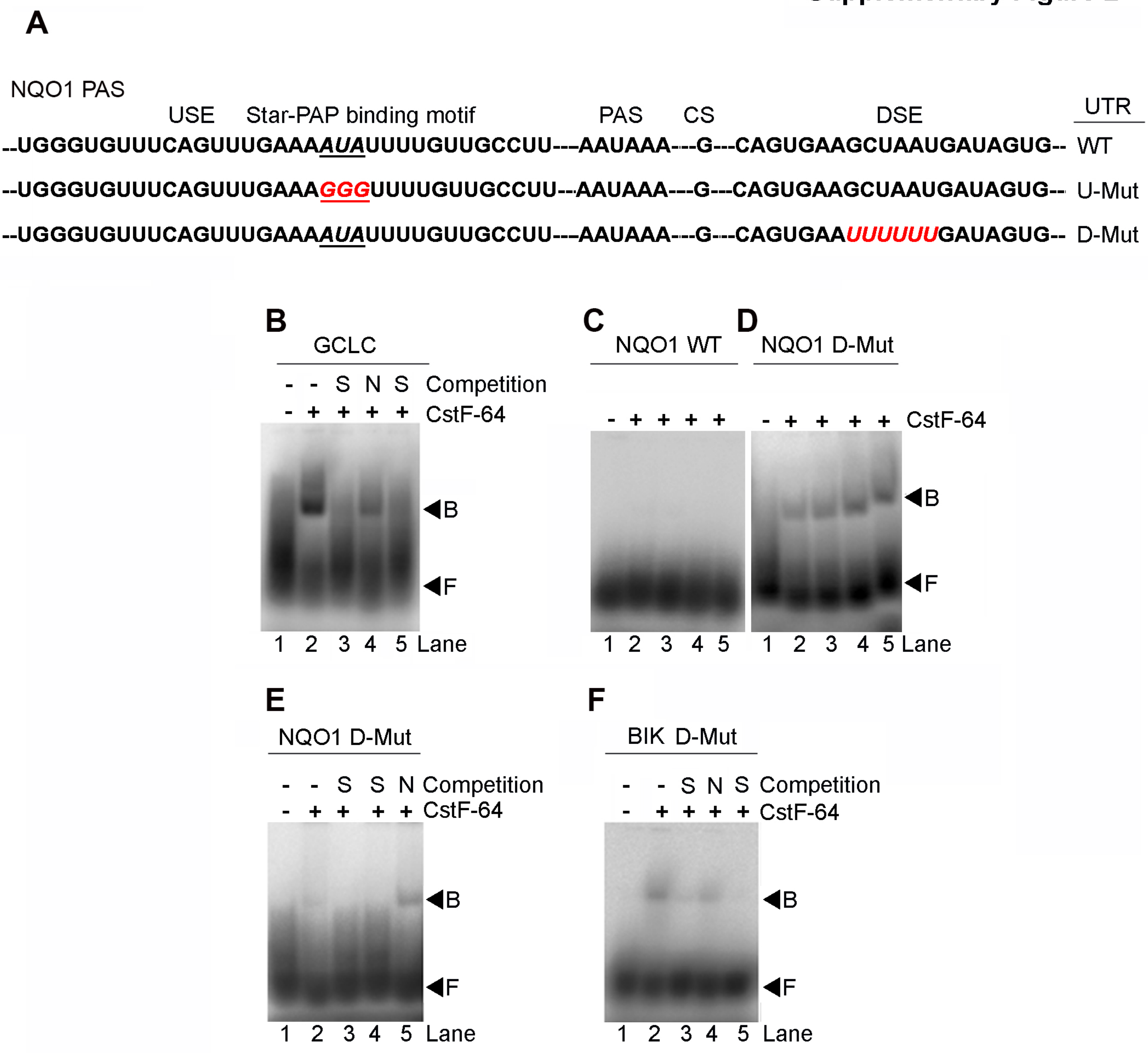

Supplementary Figure 3

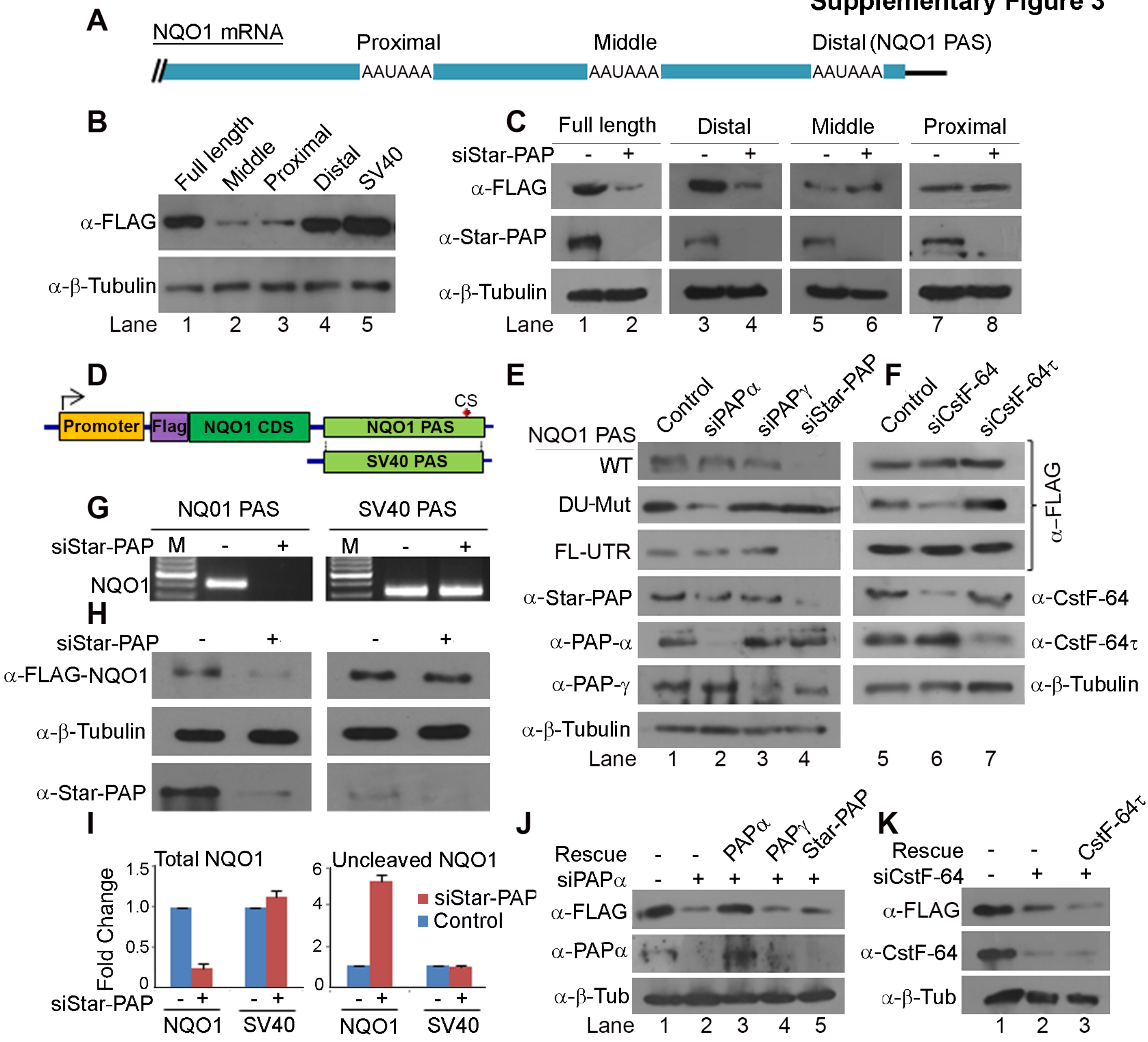

Supplementary Figure 4

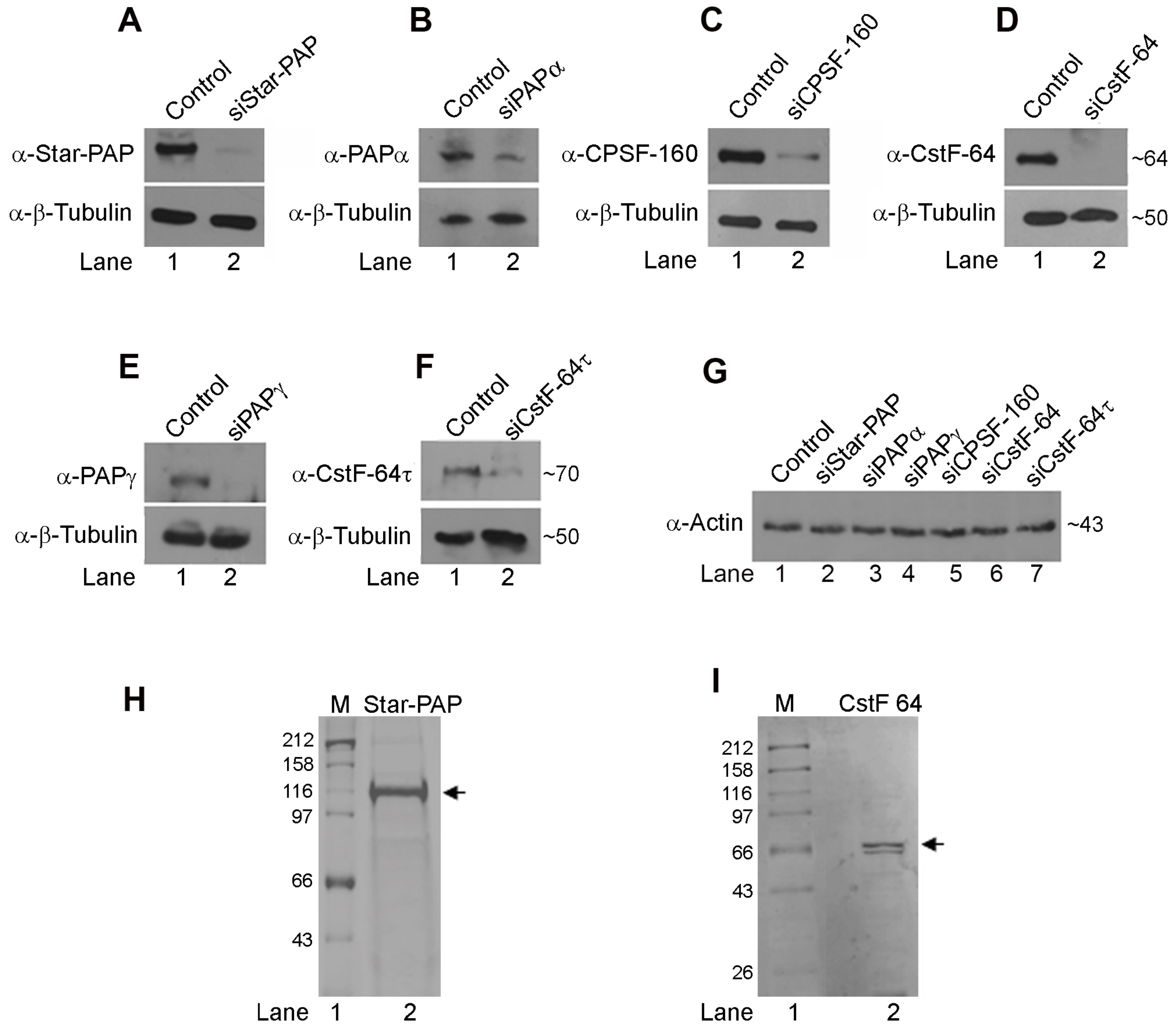

Supplementary Figure 5

A

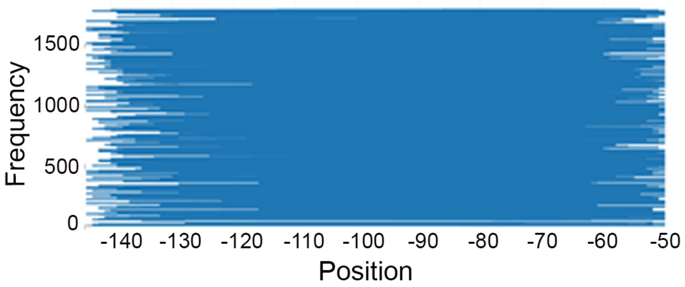

B

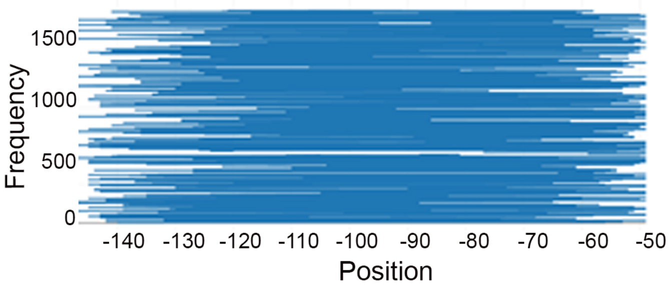

C

12 most frequently occuring 5-mers with AUA (>55%)

5-mer	p-values
UCAUA	0.00126
CAUAU	0.00126
GUAUA	0.0013
AUAUG	0.0013
UGAUA	0.0013
ACAUA	0.00133
AUACA	0.00133
UUAUA	0.00152
UAUAU	0.00152
AAUAU	0.00156
AUAUU	0.00156
AUAUA	0.0016

D

Top 15 7-mers with central -AUA- element

7-mer	p-values
GCAUAUU	0.00135
ACAUAGC	0.00193
GCAUAUA	0.00344
UCAUAUU	0.00397
UCAUAUG	0.00575
UGAUACU	0.00575
UCAUAAU	0.00641
CCAUAUA	0.00641
GGAUAUA	0.00678
UGAUAUU	0.00781
UCAUAUA	0.00781
UUAUACC	0.00781
GAAUAUU	0.00893
GUAUAUU	0.00961
ACAUAUU	0.00961
